# Tailoring the refractive index of impedance-matched ferrite composites

**DOI:** 10.1038/s41598-022-19188-3

**Published:** 2022-09-22

**Authors:** L. Parke, I. J. Youngs, C. P. Gallagher, A. P. Hibbins, J. R. Sambles

**Affiliations:** 1 Electromagnetic and Acoustic Materials Group, Department of Physics and Astronomy, University of Exeter, Stocker Road, Exeter, EX4 4QL UK; 2 Defence, Science and Technology Laboratory, Salisbury, SP4 0JQ UK

**Keywords:** Electronic devices, Sensors and biosensors, Materials science, Applied physics

## Abstract

Independent control of the magnetic and electric properties of two-part and three-part ferrite composites is demonstrated through variation of particle size and volume fraction of ferrite inclusions. This provides a route to creating broadband impedance-matched composites with tailored high refractive-index values. A two-part composite comprising NiZn ferrite in a PTFE dielectric host with approximately equal values of relative real permittivity and permeability up to 100 MHz is manufactured. The refractive index for NiZn–PTFE composites, measured at 20 MHz, is 6.1 for NiZn volume fraction of 50%vol. and 6.9 for NiZn volume fraction of 70%vol. Similarly, we have characterised a three-part composite with a refractive index of approximately 16 up to 60 MHz. The three-part composite comprises NiZn and MnZn ferrites in a PTFE dielectric host matrix with a percentage volume ratio of 65%: 15%: 20%, respectively.

## Introduction

Commercially available soft ferrites have been used extensively in telecommunications and antenna systems due to their simultaneously high real part of permeability, and low magnetic loss in the MHz frequency range^1^. The high real part of permeability not only increases the refractive index for composite materials, aiding miniaturisation, but also increases the characteristic impedance towards the impedance matched case of Z $=1.$ It is well known that the frequency dependence of the permeability diminishes at higher (GHz) frequencies due to domain wall relaxation and gyromagnetic relaxation: a phenomenon that is described by Snoek’s law^2^. Since Snoek’s original article appeared in 1948, there have been many studies extending this concept to apply to thin magnetic films and composite materials^3–5^. The original law does not account for the size or the shape of the magnetic particles if the ferrite is powdered and mixed with a host material. The size and shape of the particles and the filling fraction of the resulting composite offer additional freedoms to tune the frequency dependence of the magnetic response. For example, the shape anisotropy of magnetic inclusions can be increased by using magnetic flakes, increasing the frequency at which a strong magnetic response can be observed^6,7^. Materials with a planar crystalline geometry, such as M-type hexafarrites, have increased magnetocrystalline anisotropy, extending the frequency range of magnetic performance^8^.Of course, these same degrees of freedom also influence the dielectric (permittivity) response of the composite. In this study we demonstrate that the relative permittivity ($\varepsilon = \varepsilon^{\prime} - i\varepsilon^{\prime\prime}$) and permeability ($\mu = \mu^{\prime} - i\mu^{\prime\prime}$) of the composite are influenced by the particle size of ferrite inclusions. If the properties of the magnetic particles in composites are carefully controlled, high refractive index ($n=\sqrt{\varepsilon \mu }$) materials that are impedance matched ($Z=\sqrt{\mu /\varepsilon }$) to free space can be manufactured. These materials with high refractive index and impedance matching to free space are important for the miniaturisation of antennas.

NiZn and MnZn ferrites are magnetically ‘soft’ due to their low magnetic coercivity, meaning they do not retain magnetism after being subject to a magnetic bias. The general chemical formula for spinel ferrites is MFe_2_O_4_, where ‘M’ is a divalent metal. The spinel crystal structure, with a cubic close-packed arrangement of metallic ions surrounded by oxygen ions, leads to a high magnetocrystalline anisotropy due to the ordering of electron spins^9^. Studies of the effect of ferrite particle size and percentage volume loading of composites on the resulting complex permeability of a dielectric-based composite are not new. For example, Dosoudil et al.^10^ fabricated three sets of composite samples, using commercially available MnZn and NiZn ferrite powdered ceramics in a polyvinyl chloride (PVC) matrix. In their paper, they explore the effect of particle size on the permeability, fixing the volume loading (65% vol.) of the ferrite powder and MnZn:NiZn ratio (80%: 20%). A typical Snoek’s Law dependence was observed, characterised by a resonant peak in the imaginary component of the permeability, which shifts to higher frequencies with increasing particle size. The real part of the relative permeability ($\mu^{\prime }$) at 20 MHz increased from approximately 16 (for < 40 µm sized particles) to approximately 20 (for 80–250 µm sized particle). The increase in permeability with increasing particle size is associated with the increase in number of magnetic domains within the ferrite particles, and will be discussed later.

Slama et al.^11^ investigated the coercivity and low frequency permeability (at 100 kHz) as a function of grain size for a bulk-sintered NiZn ferrite. A linear relationship between the low frequency permeability and the grain size (for a grain size range of 2–15 µm) was observed, while an inverse relationship was found between the coercivity and the grain size. These results indicate that the intrinsic magnetic properties are significantly affected by the microstructure of the ferrite and this will be discussed later in this paper.

Of course, it is not just the magnetic properties that are affected by the ferrite particle size in composites, the dielectric properties are also altered. The effect of particle size on both the real permeability and permittivity of hexagonal ferrites has been investigated by Li et al.^12^. In that study the particle size of a 70% vol. hexagonal ferrite composite (${\mathrm{Ba}}_{3}{\mathrm{Co}}_{2}{\mathrm{Fe}}_{24}{\mathrm{O}}_{41}$) in a polyvinylidene fluoride polymer matrix was varied over the three particle size ranges; 75–150, 38–50 and 10–30 µm. At 20 MHz, the real part of the relative permeability increased from 3 to 4.8 for ferrite particle size ranges 10–30 µm and 75–150 µm, respectively, while the corresponding relative real permittivity also increased from 50 to 75. Parsons et al. also investigated how particle size affects the permeability and permittivity of polycrystalline NiZn ferrite nanoparticles in polymer composites^13^. In their study, co-precipitated NiZn nanoparticles were used to create a near impedance matched material with permittivity and permeability approximately equal to 4.5 across the frequency range 300–500 MHz. Li et al. performed studies into the particle size effects for composites comprising Co-Ti substituted M-type hexaferrite composites^14^, and presented a modified effective medium theory to predict the magnetodielectric properties. The modified effective medium theory shows magnetic permeability to have a strong dependence on particle size, particularly when the distribution of particle size is narrow.Li et al. also investigated the importance of magnetocrystalline anisotropy for M-type hexaferrites when prepared by one-step or two- step sintering procedures. By preparing hexaferrites by a two-step sintering procedure, a fine grained microstructure can be achieved with uniform domains. The uniform domains have enhanced high-frequency permeability with minimised loss. Composite samples with 89% density Co-Ti substituted barium hexaferrite in polyvinyl alcohol had a real part of permeability of 15, extending up to 500 MHz. The relative permittivity was significantly lower, at 10.8.

## Methods

In this present study, the commercially available sintered NiZn-ferrite powder and sintered MnZn-ferrite, supplied by MagDev Ltd (UK), were used to produce cold-compressed NiZn-ferrite–PTFE and NiZn-MnZn-ferrite–PTFE composites. X-ray diffraction measurement confirmed the phase compositions of NiZn ferrite to be ${\mathrm{Ni}}_{0.4}{\mathrm{Zn}}_{0.6}{\mathrm{Fe}}_{2}{\mathrm{O}}_{4}$ (with minor impurity phases of $\mathrm{NiO}$, $\mathrm{ZnO}$, ${\mathrm{Fe}}_{2}{\mathrm{O}}_{3}$ and ${\mathrm{Fe}}_{3}{\mathrm{O}}_{4})$ and MnZn ferrite to be ${\mathrm{Mn}}_{0.8}{\mathrm{Zn}}_{0.2}{\mathrm{Fe}}_{2}{\mathrm{O}}_{4}$ (with minor impurity phases of ${\mathrm{Fe}}_{3}{\mathrm{O}}_{4}$, ${\mathrm{Fe}}_{2}{\mathrm{O}}_{3}$ and MnO). The presence of impurity phases means ferrous and ferric ions exist within the bulk of the grains. These ferrous and ferric ions facilitate electron hopping across crystal sites in the grains, thus reducing the resistivity of the material and increasing the permittivity^15^.

Laser-diffraction measurements were taken using a Mastersizer2000 supplied by Malvern Instruments Ltd. (UK) gave the modal particle diameter for the NiZn ferrite powder as 4 µm. While the modal value for the particle size was below 10 µm, the range of particle sizes was between 1 and 200 µm. The values of the real part of its static relative permeability and relative permittivity as specified by MagDev Ltd. are 125 and 100. The particle size for the powder was separated into different size ranges using a vibrating sieve to produce eight size ranges: 125–90 µm; 90–75 µm; 75–63 µm; 63–53 µm; 53–45 µm; 45–38 µm; 38–20 µm and < 20 µm. The average particle size and uncertainty for each sieved fraction was measured via laser diffraction, and the size distribution deduced was found to be in good agreement with the size of the corresponding sieve-mesh.

To characterise the electromagnetic properties, composites were fabricated using each sieved sample. The NiZn ferrite powder fraction was mixed with powdered PTFE with mean particle diameter of 35 µm according to supplier, Sigma Aldrich Ltd. The powders were mixed by simply stirring the powders in a metal cylinder before sealing and shaking the mixture by hand. The powder mix was poured into a cylindrical, hardened steel mould that had been sprayed with silicone mould-release spray. The mix was then pressed in the mould under 55 MPa for 300 s to produce millable composite samples with a diameter of 30 mm and height between 5 and 10 mm. Samples were made with volume fractions of 15, 30, 50 and 70% NiZn ferrite powder (32 individual samples in total).

Similarly, a three-part composite containing 15% vol. MnZn ferrite, 65% vol. NiZn ferrite and 20% vol. PTFE was fabricated via the same method. The NiZn ferrite was used with the as-provided particle size distribution mentioned above, without any sieving. Laser diffraction measurements revealed that the MnZn ferrite powder had a median particle diameter of 35 µm, and the static values of real part of the permeability and permittivity as specified by MagDev are ${10}^{3}$ and ${10}^{5}$ respectively.

**Figure 1:**
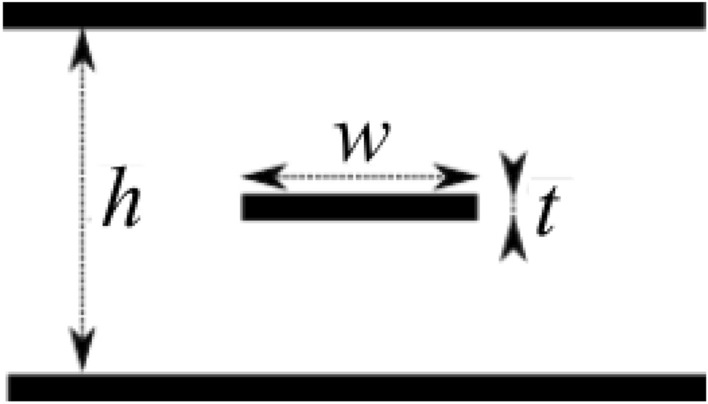
Cross-section schematic for the stripline geometry, showing width of central conductor, *w*, thickness of central conductor, *t*, and height of stripline cavity, *h*.

The samples were electromagnetically characterised using the stripline technique developed by Barry^16^. The stripline geometry had a signal line width, *w* = 19.40 mm, a signal line thickness, t = 0.10 mm, and a groundplane separation, *h* = 13.40 mm, indicated in Fig. 1. The signal line had a standard 20^O^ taper from coaxial pin to final strip width. Each of the samples were milled into two identical cuboid shapes with each of the sample pairs placed above and below the signal line to fill the transmission line cross-section. The cuboid shapes had a 0.05 mm deep slot with width 19.40 mm milled to accommodate the conducting strip of the transmission line. The stripline was connected to a Vector Network Analyser (VNA) that was calibrated using ‘Short’, ‘Open’, ‘Load’ and ‘Through’ standard calibration (SOLT calibration) to set the reflection planes to the ends of coaxial cables. The complex reflection and transmission coefficients of the samples was measured by inserting the ferrite samples into the stripline and measuring the complex S_11_ and S_21_ parameters. From these complex S-parameters together with the frequency and the thickness of the sample, the relative complex permittivity and permeability are both obtained using the Nicholson, Ross, Weir (NRW) extraction method^17,18^.

## Results

**Figure 2:**
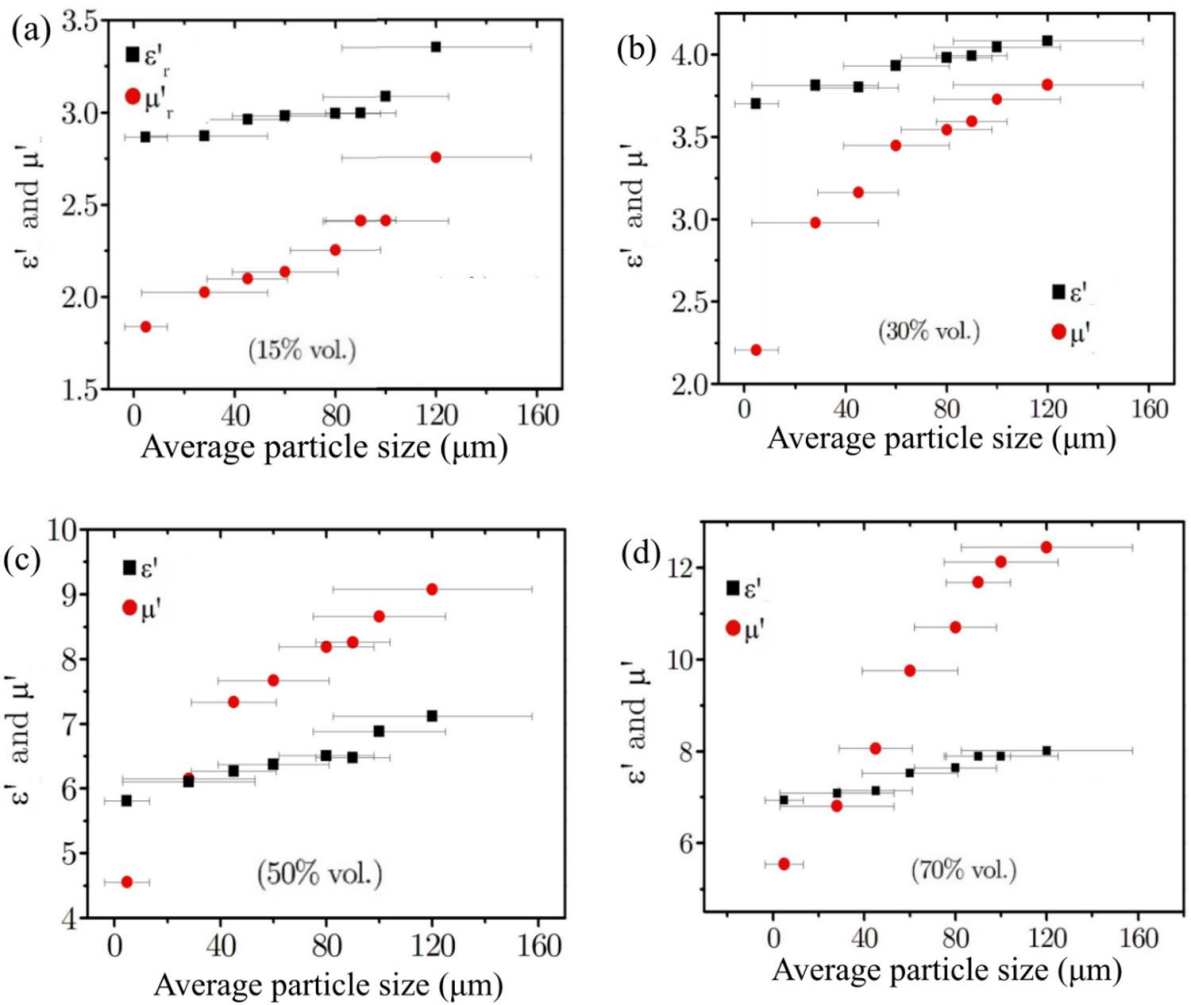
Plots of the relative real permittivity (*ε*ˊ) and permeability (*μ*ˊ) at 20 MHz as a function of the average NiZn ferrite particle size for a NiZn ferrite-PTFE composite with (**a**) 15% vol., (**b**) 30% vol., (**c**) 50% vol. and (**d**) 70% vol. loading of ferrite material. The average particle size and uncertainty were deduced from laser diffraction measurements.

Figure 2 shows the real parts of the relative permittivity and permeability at 20 MHz as a function of the average NiZn ferrite particle size for a NiZn ferrite-PTFE composite for (a) 15% vol., (b) 30% vol., (c) 50% vol. and (d) 70% vol. All four plots show that both the permeability and permittivity increase as a function of the NiZn ferrite particle size; however, the permeability is more strongly dependent on the particle size in comparison to the permittivity. For example, in Fig. 2d, the permittivity increases from 7.0 (for an average particle size of 4.2 µm) to 7.9 (for an average particle size of 147 µm) while the corresponding permeability value increases from 5.5 to 12.5 for the same particle size increase. The increase in permeability with increased particle size can be understood by considering the distribution of the demagnetising fields for particles containing differing numbers of domains.

Studies into the domain structure of MnZn ferrite have found that mono-domains exist in grains that are approximately 4 µm or less in size^19^. The domain structure and number of domains is dictated by the internal energy of the particle with the magnetostatic energy and the exchange energy competing. The magnetostatic energy is minimised by division of the particle into more domains, reducing domain size, while the exchange energy is minimised by aligning adjacent spins, increasing domain size. As the particle subdivides into more domains, the coercivity decreases as less magnetic force is required to move domain walls than to rotate the magnetisation vectors of entire domains. In general, magnetic materials with a low coercivity value possess a large permeability^20^ as they can be magnetised more easily.

Figure 2 also shows a weak dependence of the permittivity on particle size. This can be understood by considering the grain structure within each of the ferrite particles. The dielectric properties of ferrites originate from their granular structure, being composed of semi-conducting grains. An electron hopping process takes place across crystal lattice sites within the grains that have ferric and ferrous ions^15^. In grain boundaries, impurity ions replace the ferrous ions and suppress the hopping effect, increasing resistivity^21^. A capacitive effect between internal barrier layers (IBLC) can be manifest in Mn–Zn ferrites, leading to a ‘giant dielectric phenomenon’^22,23^. It has been documented that the presence of the grain boundaries greatly increases the relative permittivity of the ferrite, as capacitive effects arising from the separation of the charges enhance the permittivity^24^. The larger ferrite particles in this study are composed of more grains and insulating grain boundaries compared to smaller ferrite particles. In some cases, very small particles less than 10 µm can be mono-grain^25^. Therefore, the permittivity of the smaller ferrite particles is reduced since the capacitive effects arising from the grain boundaries, are reduced.

Figure 2 demonstrates that for the more heavily loaded composites, 50–70% vol., equal values of the real parts of the relative permittivity and permeability can be obtained by tuning the ferrite-particle size between 25 and30 μm. The corresponding refractive index values of the composites are 6.1 (50% vol.) and 6.9 (70% vol.) respectively at 20 MHz. If the ferrite composite has low electromagnetic loss, this offers the exciting prospect of a high-index, impedance matched material.

**Figure 3:**
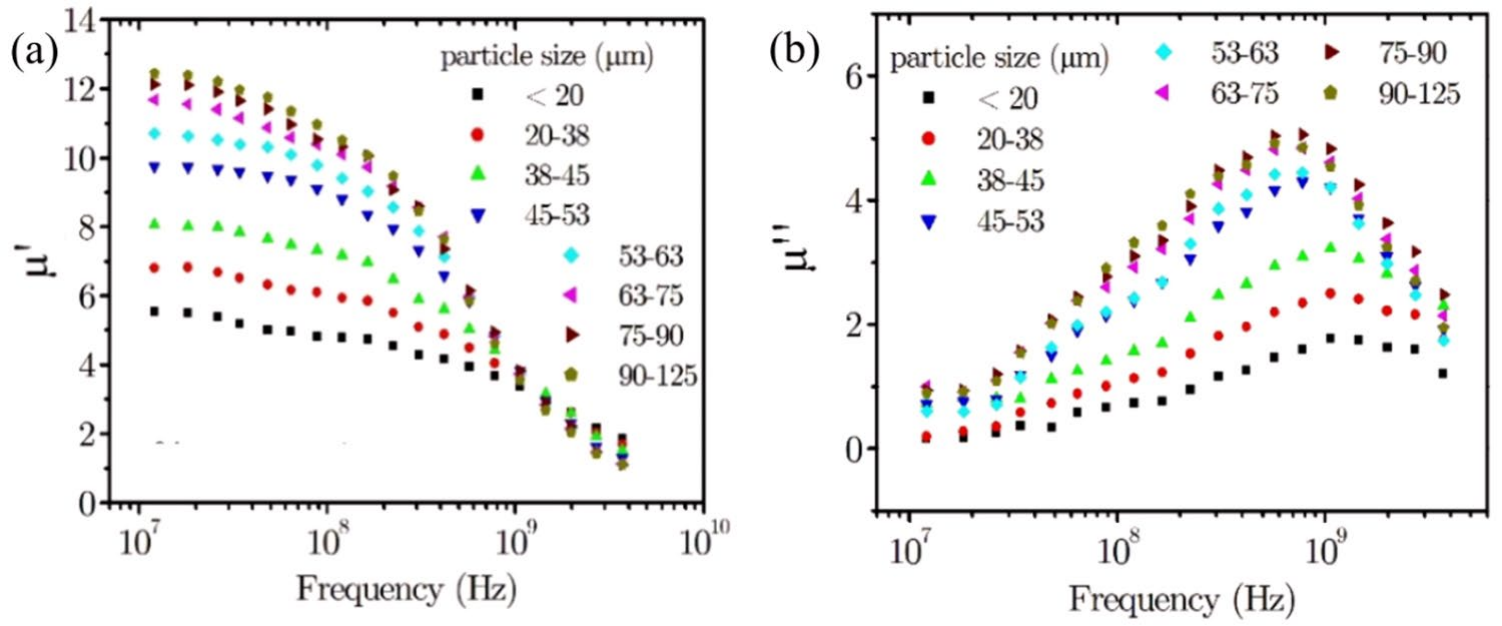
Plots of the complex relative permeability as a function of frequency for eight 70% vol. NiZn ferrite-PTFE composites containing different particle size ranges: (**a**) the real part of the relative permeability, while (**b**) the imaginary permeability.

Figure 3 shows the complex relative permeability as a function of frequency for eight 70% vol. NiZn ferrite-PTFE composites containing different particle size ranges, showing (a) the real part, and (b) the imaginary part of the relative permeability. These plots show that the change in the frequency dispersion of the complex permeability for composites containing different particle sizes follows Snoek’s Law^2^. That is, the relative permeability falls off faster with frequency when the initial value for the real part is higher. The frequency dispersion of the relative permeability behaves in a similar manner to that of the complex permeability for composites containing different volume fractions of ferrite filler where larger particles give a larger initial relative permeability, similar to a higher volume fraction of ferrite loading.

**Figure 4:**
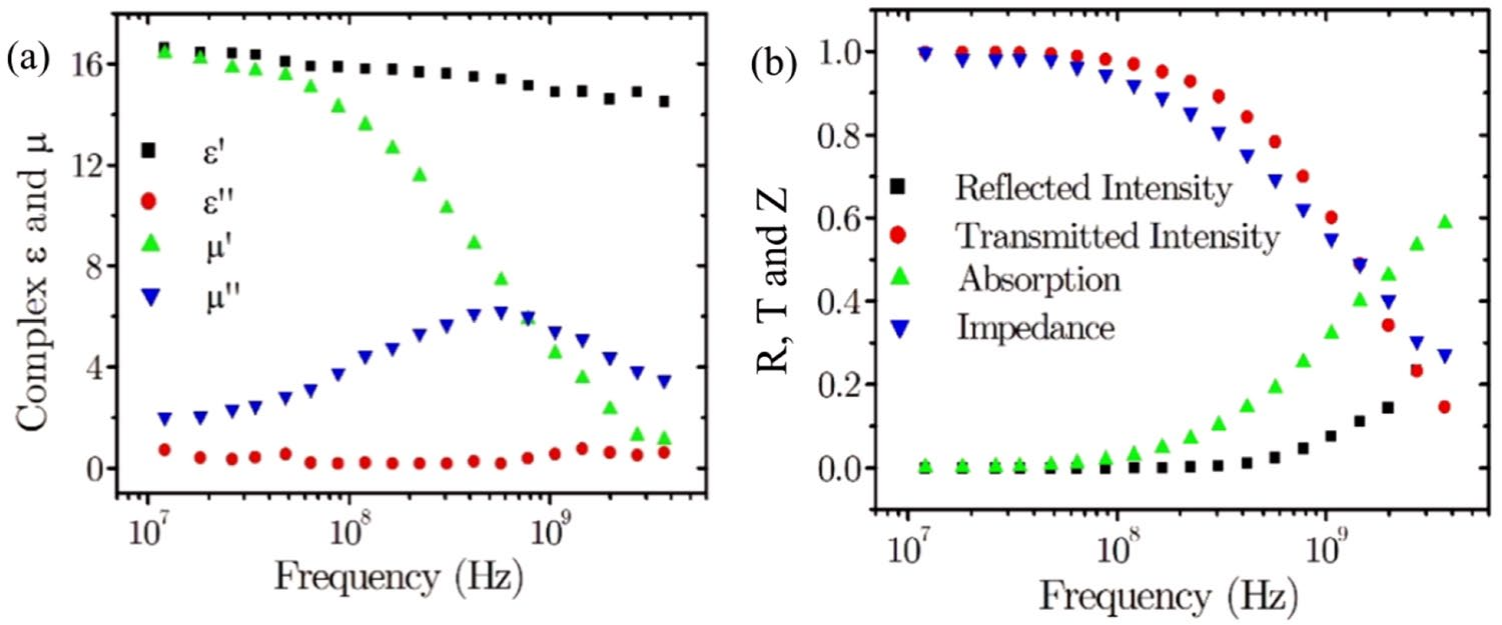
(**a**) Plot of the complex relative permeability and permittivity as a function of frequency and (**b**) reflected and transmitted intensity, absorption and the relative impedance as a function of frequency for a 65% vol. NiZn ferrite, 15% vol. MnZn ferrite and 20% vol. PTFE composite.

In the final part of this study, a three-part composite containing MnZn ferrite, NiZn ferrite and PTFE is fabricated as an alternative approach to engineering impedance matched, high-index materials. The proportions of each component were chosen to provide the highest possible refractive index of the composite under the assumption that samples can be fabricated with a maximum ferrite loading volume fraction of 80% vol. PTFE powder was used as the dielectric matrix. Figure 4a shows the frequency dependent complex permittivity and permeability for a composite containing 65% vol. NiZn ferrite (modal particle size 4 µm), 15% vol. MnZn ferrite (modal particle size 35 µm) and 20% vol. PTFE (particle size < 35 µm). For this part of the study, the powders are used in the as-received state directly from the manufacturer. That is, the particles sizes for the ferrite inclusions did not undergo any secondary sieving stage. The real parts of the permittivity and permeability are closely matched below 100 MHz, with the relative impedance falling to 94% of its low-frequency value at 100 MHz (Fig. 4b). The measured transmissivity is near complete (> 98%) with the refractive index at n ~ 16. With a higher index dielectric host material, higher values for refractive index may be obtained. The PTFE host material has a dielectric permittivity of around 2.2, corresponding to a refractive index of 1.48^26^. Above 100 MHz there is an increasing mismatch of complex permittivity and permeability associated with the domain wall relaxation^27,28^. At 4 GHz, the absorbance increases to 60% due to the increase in the imaginary part of permeability, while the relative impedance decreases to 0.28 due to the reduction of the real part of permeability.

## Conclusions

In conclusion, both the relative real permeability and permittivity for NiZn ferrite-PTFE composites were found to increase with increasing ferrite particle size. The permeability exhibited a far stronger particle size dependence in comparison to the permittivity, allowing an impedance matched condition to be met at 20 MHz for particle sizes of 25–30 µm with both 50 and 70% vol. NiZn ferrite composites. The corresponding refractive index values of these two composites are 6.1 and 6.9 respectively. A third component, MnZn ferrite, added to NiZn ferrite-PTFE composites allows the relative permittivity and permeability to both equal 16.1 from10 to 50 MHz. The ability to alter the permeability and permittivity by controlling the particle size in magnetic composites or introducing a third component allows for desired EM properties, such as broadband impedance matching to free space to be engineered. Components requiring high refractive index, low electromagnetic loss materials, such as lenses and miniaturised antennas will benefit from these composites.

## Supplementary Information

Supplementary Information 1.

Supplementary Information 2.

Supplementary Information 3.

Supplementary Information 4.

## Data Availability

The datasets generated and/or analysed during the current study are available in the University of Exeter repository, Open Repository Exeter (ORE), reference^29^ gives a web link for this repository.
