# Supplementary material for: Tailoring the refractive index of impedance-matched ferrite composites
Source: Sci Rep. 2022 Sep 22;12:15818. doi: 10.1038/s41598-022-19188-3 (PMC9500025; doi:10.1038/s41598-022-19188-3)
Supplement: Supplementary file 1 — Supplementary Information 1. [file 41598_2022_19188_MOESM1_ESM.zip › 41598_2022_19188_MOESM1/125-93_Ave.pdf]

MASTERSIZER 2000

Result Analysis Report

Sample Name:
125-93 - Average

SOP Name:
NiZn ferrite

Measured:
21 January 2014 12:52:16

Sample Source & type:
Paris

Measured by:
Mastersizer 2000

Analysed:
21 January 2014 12:52:18

Sample bulk lot ref:
123-ABC

Result Source:
Averaged

Particle Name:
NiZn ferrite

Accessory Name:
Hydro 2000MU (A)

Analysis model:
General purpose

Sensitivity:
Enhanced

Particle RI:
2.730

Absorption:
10

Size range:
0.020 to 2000.000 um

Obscuration:
11.25 %

Dispersant Name:
Water

Dispersant RI:
1.330

Weighted Residual:
0.788 %

Result Emulation:
Off

Concentration:
0.1846 %Vol

Span :
0.802

Uniformity:
0.264

Result units:
Volume

Specific Surface Area:
0.0584 m²/g

Surface Weighted Mean D[3,2]:
102.788 um

Vol. Weighted Mean D[4,3]:
139.451 um

d(0.1): 89.528 um **d(0.5): 132.432 um** **d(0.9): 195.722 um**

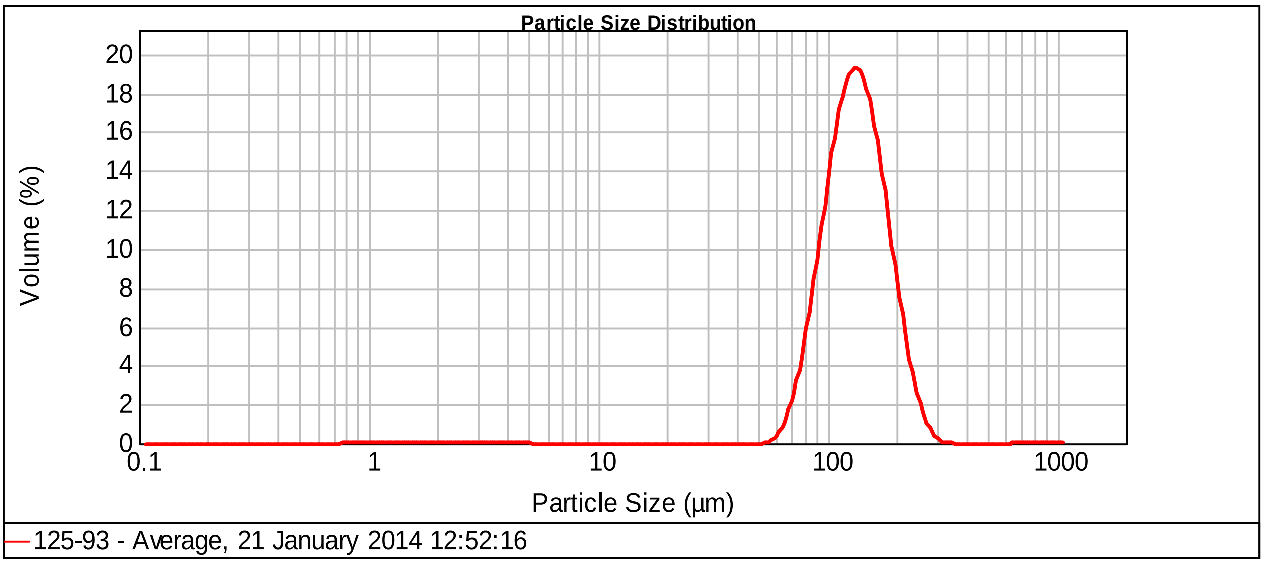

Size (μm)	Volume In %
0.010	0.00
0.011	0.00
0.013	0.00
0.015	0.00
0.017	0.00
0.020	0.00
0.023	0.00
0.026	0.00
0.030	0.00
0.035	0.00
0.040	0.00
0.046	0.00
0.052	0.00
0.060	0.00
0.069	0.00
0.079	0.00
0.091	0.00
0.105	

Size (μm)	Volume In %
0.105	0.00
0.120	0.00
0.138	0.00
0.158	0.00
0.182	0.00
0.209	0.00
0.240	0.00
0.275	0.00
0.316	0.00
0.363	0.00
0.417	0.00
0.479	0.00
0.550	0.00
0.631	0.00
0.724	0.01
0.832	0.02
0.955	0.02
1.096	

Size (μm)	Volume In %
1.096	0.02
1.259	0.03
1.445	0.03
1.660	0.03
1.905	0.03
2.188	0.03
2.512	0.03
2.884	0.03
3.311	0.03
3.802	0.02
4.365	0.01
5.012	0.00
5.754	0.00
6.607	0.00
7.586	0.00
8.710	0.00
10.000	0.00
11.482	

Size (μm)	Volume In %
11.482	0.00
13.183	0.00
15.136	0.00
17.378	0.00
19.953	0.00
22.909	0.00
26.303	0.00
30.200	0.00
34.674	0.00
39.811	0.00
45.709	0.00
52.481	0.09
60.256	0.92
69.183	3.07
79.433	6.75
91.201	11.30
104.713	15.39
120.226	

Size (μm)	Volume In %
120.226	17.41
138.038	16.52
158.489	13.14
181.970	8.55
208.930	4.43
239.883	1.62
275.423	0.30
316.228	0.00
363.078	0.00
416.869	0.00
478.630	0.00
549.541	0.00
630.957	0.00
724.436	0.03
831.764	0.07
954.993	0.08
1096.478	0.00
1258.925	

Size (μm)	Volume In %
1258.925	0.00
1445.440	0.00
1659.587	0.00
1905.461	0.00
2187.762	0.00
2511.886	0.00
2884.032	0.00
3311.311	0.00
3801.894	0.00
4365.158	0.00
5011.872	0.00
5754.399	0.00
6606.934	0.00
7585.776	0.00
8709.636	0.00
10000.000	

Operator notes:
