# Supplementary material for: Tailoring the refractive index of impedance-matched ferrite composites
Source: Sci Rep. 2022 Sep 22;12:15818. doi: 10.1038/s41598-022-19188-3 (PMC9500025; doi:10.1038/s41598-022-19188-3)
Supplement: Supplementary file 1 — Supplementary Information 1. [file 41598_2022_19188_MOESM1_ESM.zip › 41598_2022_19188_MOESM1/20_Ave.pdf]

MASTERSIZER 2000

Result Analysis Report

**Sample Name:**<br><20 - Average	**SOP Name:**<br>NiZn ferrite	**Measured:**<br>21 January 2014 13:18:24
**Sample Source & type:**<br>Paris	**Measured by:**<br>Mastersizer 2000	**Analysed:**<br>21 January 2014 13:18:25
**Sample bulk lot ref:**<br>123-ABC	**Result Source:**<br>Averaged	

**Particle Name:**<br>NiZn ferrite	**Accessory Name:**<br>Hydro 2000MU (A)	**Analysis model:**<br>General purpose	**Sensitivity:**<br>Enhanced
**Particle RI:**<br>2.730	**Absorption:**<br>10	**Size range:**<br>0.020 to 2000.000 um	**Obscuration:**<br>11.45 %
**Dispersant Name:**<br>Water	**Dispersant RI:**<br>1.330	**Weighted Residual:**<br>0.662 %	**Result Emulation:**<br>Off

**Concentration:**<br>0.0054 %Vol	**Span :**<br>2.486	**Uniformity:**<br>0.769	**Result units:**<br>Volume
**Specific Surface Area:**<br>1.67 m²/g	**Surface Weighted Mean D[3,2]:**<br>3.584 um	**Vol. Weighted Mean D[4,3]:**<br>6.242 um	

d(0.1): 1.767 um **d(0.5): 4.563 um** **d(0.9): 13.109 um**

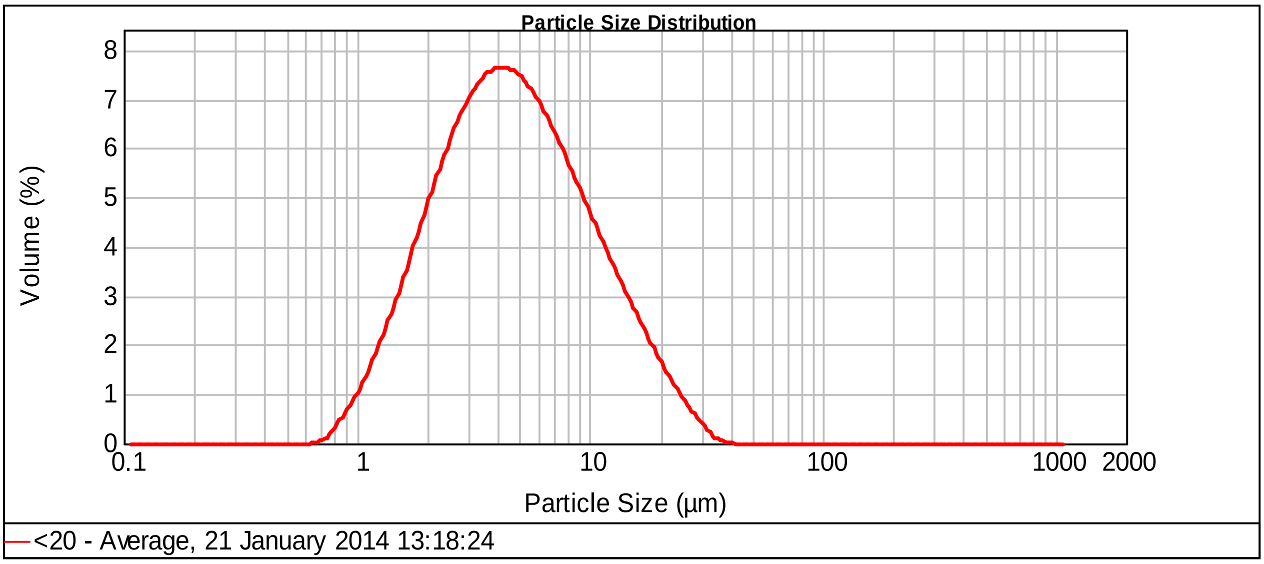

Size (µm)	Volume In %	Size (µm)	Volume In %	Size (µm)	Volume In %	Size (µm)	Volume In %	Size (µm)	Volume In %	Size (µm)	Volume In %
0.010	0.00	0.105	0.00	1.096	1.54	11.482	3.38	120.226	0.00	1258.925	0.00
0.011	0.00	0.120	0.00	1.259	2.19	13.183	2.82	138.038	0.00	1445.440	0.00
0.013	0.00	0.138	0.00	1.445	2.92	15.136	2.29	158.489	0.00	1659.587	0.00
0.015	0.00	0.158	0.00	1.660	3.70	17.378	1.78	181.970	0.00	1905.461	0.00
0.017	0.00	0.182	0.00	1.905	4.48	19.953	1.31	208.930	0.00	2187.762	0.00
0.020	0.00	0.209	0.00	2.188	5.23	22.909	0.88	239.883	0.00	2511.886	0.00
0.023	0.00	0.240	0.00	2.512	5.89	26.303	0.53	275.423	0.00	2884.032	0.00
0.026	0.00	0.275	0.00	2.884	6.41	30.200	0.22	316.228	0.00	3311.311	0.00
0.030	0.00	0.316	0.00	3.311	6.75	34.674	0.05	363.078	0.00	3801.894	0.00
0.035	0.00	0.363	0.00	3.802	6.90	39.811	0.00	416.869	0.00	4365.158	0.00
0.040	0.00	0.417	0.00	4.365	6.84	45.709	0.00	478.630	0.00	5011.872	0.00
0.046	0.00	0.479	0.00	5.012	6.60	52.481	0.00	549.541	0.00	5754.399	0.00
0.052	0.00	0.550	0.00	5.754	6.20	60.256	0.00	630.957	0.00	6606.934	0.00
0.060	0.00	0.631	0.03	6.607	5.70	69.183	0.00	724.436	0.00	7585.776	0.00
0.069	0.00	0.724	0.19	7.586	5.13	79.433	0.00	831.764	0.00	8709.636	0.00
0.079	0.00	0.832	0.57	8.710	4.54	91.201	0.00	954.993	0.00	10000.000	
0.091	0.00	0.955	0.99	10.000	3.95	104.713	0.00	1096.478	0.00		
0.105		1.096		11.482		120.226		1258.925			

Operator notes:
