# Supplementary material for: Tailoring the refractive index of impedance-matched ferrite composites
Source: Sci Rep. 2022 Sep 22;12:15818. doi: 10.1038/s41598-022-19188-3 (PMC9500025; doi:10.1038/s41598-022-19188-3)
Supplement: Supplementary file 1 — Supplementary Information 1. [file 41598_2022_19188_MOESM1_ESM.zip › 41598_2022_19188_MOESM1/38-20_Ave.pdf]

MASTERSIZER 2000

Result Analysis Report

Sample Name:
38-20 - Average

SOP Name:
NiZn ferrite

Measured:
21 January 2014 13:13:43

Sample Source & type:
Paris

Measured by:
Mastersizer 2000

Analysed:
21 January 2014 13:13:44

Sample bulk lot ref:
123-ABC

Result Source:
Averaged

Particle Name:
NiZn ferrite

Accessory Name:
Hydro 2000MU (A)

Analysis model:
General purpose

Sensitivity:
Enhanced

Particle RI:
2.730

Absorption:
10

Size range:
0.020 to 2000.000 um

Obscuration:
10.48 %

Dispersant Name:
Water

Dispersant RI:
1.330

Weighted Residual:
1.033 %

Result Emulation:
Off

Concentration:
0.0085 %Vol

Span :
2.613

Uniformity:
0.832

Result units:
Volume

Specific Surface Area:
0.983 m²/g

Surface Weighted Mean D[3,2]:
6.103 um

Vol. Weighted Mean D[4,3]:
15.827 um

d(0.1): **2.404** **um** **d(0.5):** **12.432** **um** **d(0.9):** **34.894** **um**

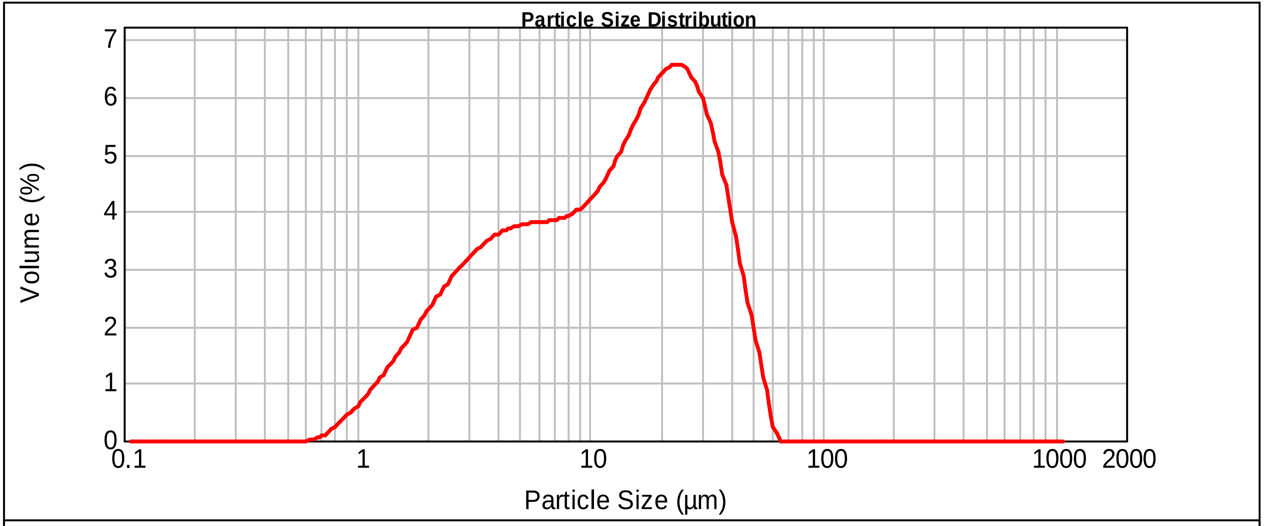

—38-20 - Average, 21 January 2014 13:13:43

Size (µm)	Volume In %	Size (µm)	Volume In %	Size (µm)	Volume In %	Size (µm)	Volume In %	Size (µm)	Volume In %	Size (µm)	Volume In %
0.010	0.00	0.105	0.00	1.096	0.84	11.482	4.27	120.226	0.00	1258.925	0.00
0.011	0.00	0.120	0.00	1.259	1.13	13.183	4.70	138.038	0.00	1445.440	0.00
0.013	0.00	0.138	0.00	1.445	1.44	15.136	5.16	158.489	0.00	1659.587	0.00
0.015	0.00	0.158	0.00	1.660	1.76	17.378	5.58	181.970	0.00	1905.461	0.00
0.017	0.00	0.182	0.00	1.905	2.08	19.953	5.86	208.930	0.00	2187.762	0.00
0.020	0.00	0.209	0.00	2.188	2.39	22.909	5.91	239.883	0.00	2511.886	0.00
0.023	0.00	0.240	0.00	2.512	2.67	26.303	5.65	275.423	0.00	2884.032	0.00
0.026	0.00	0.275	0.00	2.884	2.92	30.200	5.07	316.228	0.00	3311.311	0.00
0.030	0.00	0.316	0.00	3.311	3.11	34.674	4.19	363.078	0.00	3801.894	0.00
0.035	0.00	0.363	0.00	3.802	3.26	39.811	3.11	416.869	0.00	4365.158	0.00
0.040	0.00	0.417	0.00	4.365	3.36	45.709	1.96	478.630	0.00	5011.872	0.00
0.046	0.00	0.479	0.00	5.012	3.41	52.481	0.91	549.541	0.00	5754.399	0.00
0.052	0.00	0.550	0.00	5.754	3.44	60.256	0.03	630.957	0.00	6606.934	0.00
0.060	0.00	0.631	0.05	6.607	3.47	69.183	0.00	724.436	0.00	7585.776	0.00
0.069	0.00	0.724	0.16	7.586	3.54	79.433	0.00	831.764	0.00	8709.636	0.00
0.079	0.00	0.832	0.37	8.710	3.68	91.201	0.00	954.993	0.00	10000.000	
0.091	0.00	0.955	0.58	10.000	3.93	104.713	0.00	1096.478	0.00		
0.105		1.096		11.482		120.226		1258.925			

Operator notes:
