# Supplementary material for: Tailoring the refractive index of impedance-matched ferrite composites
Source: Sci Rep. 2022 Sep 22;12:15818. doi: 10.1038/s41598-022-19188-3 (PMC9500025; doi:10.1038/s41598-022-19188-3)
Supplement: Supplementary file 1 — Supplementary Information 1. [file 41598_2022_19188_MOESM1_ESM.zip › 41598_2022_19188_MOESM1/45-38_Ave.pdf]

MASTERSIZER 2000

Result Analysis Report

Sample Name:
45-38 - Average

SOP Name:
NiZn ferrite

Measured:
21 January 2014 13:09:01

Sample Source & type:
Paris

Measured by:
Mastersizer 2000

Analysed:
21 January 2014 13:09:02

Sample bulk lot ref:
123-ABC

Result Source:
Averaged

Particle Name:
NiZn ferrite

Accessory Name:
Hydro 2000MU (A)

Analysis model:
General purpose

Sensitivity:
Enhanced

Particle RI:
2.730

Absorption:
10

Size range:
0.020 to 2000.000 um

Obscuration:
9.93 %

Dispersant Name:
Water

Dispersant RI:
1.330

Weighted Residual:
0.341 %

Result Emulation:
Off

Concentration:
0.0252 %Vol

Span :
0.847

Uniformity:
0.289

Result units:
Volume

Specific Surface Area:
0.319 m²/g

Surface Weighted Mean D[3,2]:
18.824 um

Vol. Weighted Mean D[4,3]:
40.774 um

d(0.1): 24.970 um **d(0.5): 40.831 um** **d(0.9): 59.537 um**

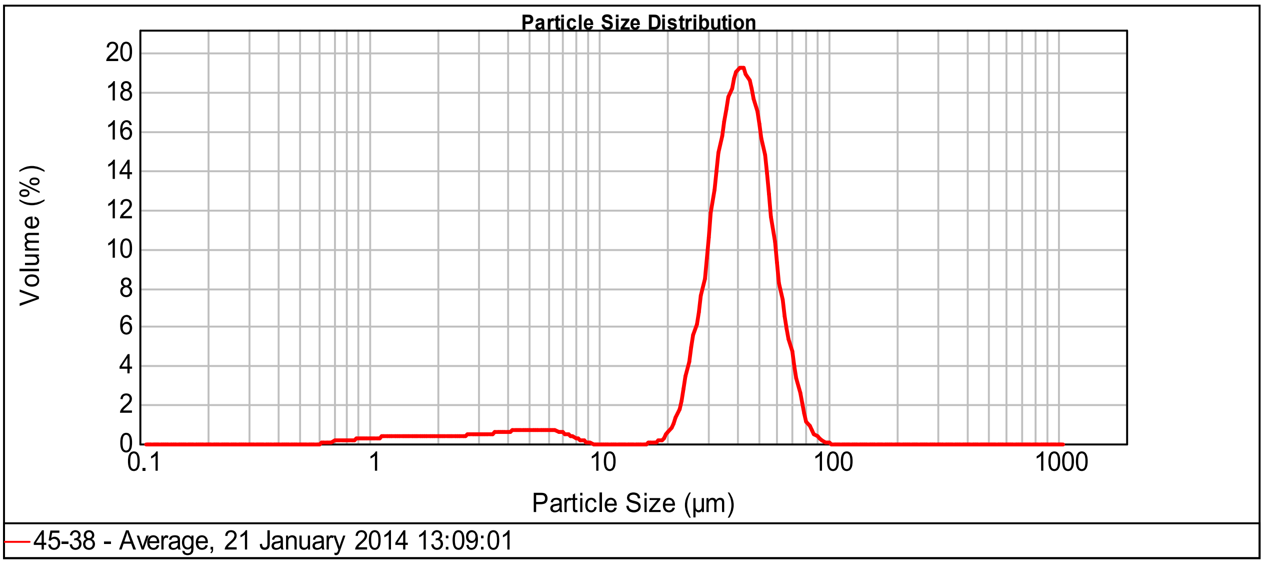

Size (μm)	Volume In %
0.010	0.00
0.011	0.00
0.013	0.00
0.015	0.00
0.017	0.00
0.020	0.00
0.023	0.00
0.026	0.00
0.030	0.00
0.035	0.00
0.040	0.00
0.046	0.00
0.052	0.00
0.060	0.00
0.069	0.00
0.079	0.00
0.091	0.00
0.105	

Size (μm)	Volume In %
0.105	0.00
0.120	0.00
0.138	0.00
0.158	0.00
0.182	0.00
0.209	0.00
0.240	0.00
0.275	0.00
0.316	0.00
0.363	0.00
0.417	0.00
0.479	0.00
0.550	0.00
0.631	0.07
0.724	0.14
0.832	0.20
0.955	0.25
1.096	

Size (μm)	Volume In %
1.096	0.30
1.259	0.33
1.445	0.35
1.660	0.36
1.905	0.36
2.188	0.37
2.512	0.38
2.884	0.42
3.311	0.48
3.802	0.56
4.365	0.63
5.012	0.66
5.754	0.63
6.607	0.48
7.586	0.22
8.710	0.00
10.000	0.00
11.482	

Size (μm)	Volume In %
11.482	0.00
13.183	0.00
15.136	0.00
17.378	0.15
19.953	0.91
22.909	3.53
26.303	6.80
30.200	12.24
34.674	15.99
39.811	17.40
45.709	15.38
52.481	11.13
60.256	5.93
69.183	2.71
79.433	0.59
91.201	0.05
104.713	0.00
120.226	

Size (μm)	Volume In %
120.226	0.00
138.038	0.00
158.489	0.00
181.970	0.00
208.930	0.00
239.883	0.00
275.423	0.00
316.228	0.00
363.078	0.00
416.869	0.00
478.630	0.00
549.541	0.00
630.957	0.00
724.436	0.00
831.764	0.00
954.993	0.00
1096.478	0.00
1258.925	

Size (μm)	Volume In %
1258.925	0.00
1445.440	0.00
1659.587	0.00
1905.461	0.00
2187.762	0.00
2511.886	0.00
2884.032	0.00
3311.311	0.00
3801.894	0.00
4365.158	0.00
5011.872	0.00
5754.399	0.00
6606.934	0.00
7585.776	0.00
8709.636	0.00
10000.000	

Operator notes:
