# Supplementary material for: Tailoring the refractive index of impedance-matched ferrite composites
Source: Sci Rep. 2022 Sep 22;12:15818. doi: 10.1038/s41598-022-19188-3 (PMC9500025; doi:10.1038/s41598-022-19188-3)
Supplement: Supplementary file 1 — Supplementary Information 1. [file 41598_2022_19188_MOESM1_ESM.zip › 41598_2022_19188_MOESM1/63-53_Ave.pdf]

MASTERSIZER 2000

Result Analysis Report

Sample Name:
63-53 - Average

Sample Source & type:
Paris

Sample bulk lot ref:
123-ABC

SOP Name:
NiZn ferrite

Measured by:
Mastersizer 2000

Result Source:
Averaged

Measured:
21 January 2014 12:46:54

Analysed:
21 January 2014 12:46:55

Particle Name:
NiZn ferrite

Particle RI:
2.730

Dispersant Name:
Water

Accessory Name:
Hydro 2000MU (A)

Absorption:
10

Dispersant RI:
1.330

Analysis model:
General purpose

Size range:
0.020 to 2000.000 um

Weighted Residual:
0.676 %

Sensitivity:
Enhanced

Obscuration:
10.61 %

Result Emulation:
Off

Concentration:
0.1127 %Vol

Specific Surface Area:
0.082 m²/g

Span :
0.756

Surface Weighted Mean D[3,2]:
73.147 um

Uniformity:
0.234

Vol. Weighted Mean D[4,3]:
79.296 um

Result units:
Volume

d(0.1): 52.641 um **d(0.5): 76.152 um** **d(0.9): 110.177 um**

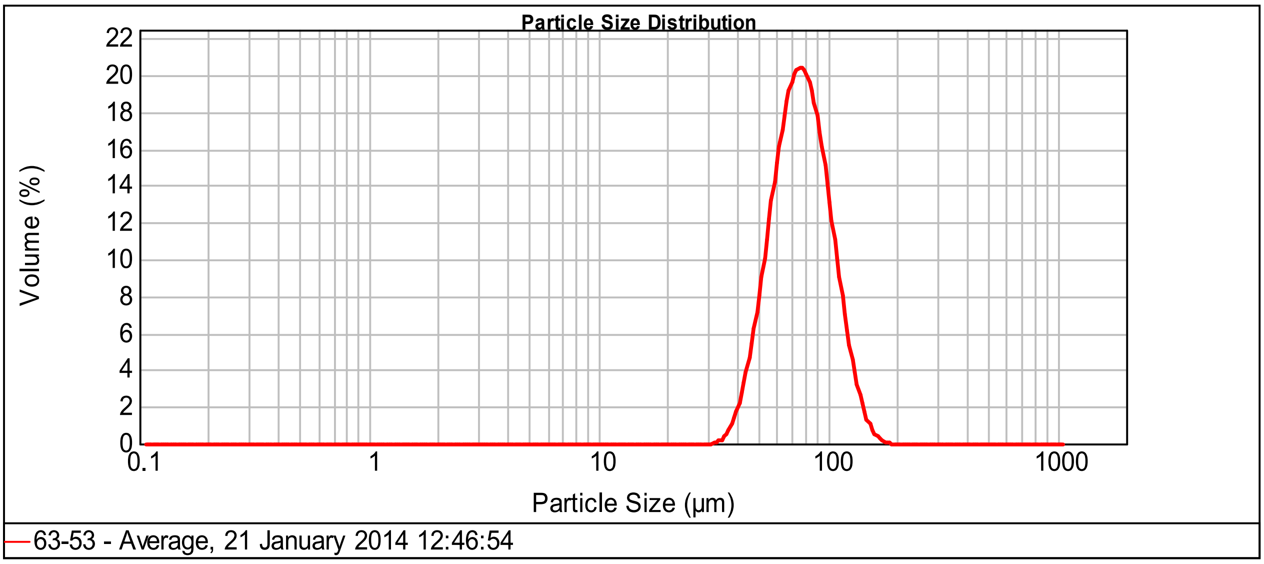

Size (μm)	Volume In %
0.010	0.00
0.011	0.00
0.013	0.00
0.015	0.00
0.017	0.00
0.020	0.00
0.023	0.00
0.026	0.00
0.030	0.00
0.035	0.00
0.040	0.00
0.046	0.00
0.052	0.00
0.060	0.00
0.069	0.00
0.079	0.00
0.091	0.00
0.105	

Size (μm)	Volume In %
0.105	0.00
0.120	0.00
0.138	0.00
0.158	0.00
0.182	0.00
0.209	0.00
0.240	0.00
0.275	0.00
0.316	0.00
0.363	0.00
0.417	0.00
0.479	0.00
0.550	0.00
0.631	0.00
0.724	0.00
0.832	0.00
0.955	0.00
1.096	

Size (μm)	Volume In %
1.096	0.00
1.259	0.00
1.445	0.00
1.660	0.00
1.905	0.00
2.188	0.00
2.512	0.00
2.884	0.00
3.311	0.00
3.802	0.00
4.365	0.00
5.012	0.00
5.754	0.00
6.607	0.00
7.586	0.00
8.710	0.00
10.000	0.00
11.482	

Size (μm)	Volume In %
11.482	0.00
13.183	0.00
15.136	0.00
17.378	0.00
19.953	0.00
22.909	0.00
26.303	0.00
30.200	0.03
34.674	0.69
39.811	2.67
45.709	6.41
52.481	11.40
60.256	16.06
69.183	18.40
79.433	17.33
91.201	13.37
104.713	8.25
120.226	

Size (μm)	Volume In %
120.226	3.93
138.038	1.27
158.489	0.18
181.970	0.00
208.930	0.00
239.883	0.00
275.423	0.00
316.228	0.00
363.078	0.00
416.869	0.00
478.630	0.00
549.541	0.00
630.957	0.00
724.436	0.00
831.764	0.00
954.993	0.00
1096.478	0.00
1258.925	

Size (μm)	Volume In %
1258.925	0.00
1445.440	0.00
1659.587	0.00
1905.461	0.00
2187.762	0.00
2511.886	0.00
2884.032	0.00
3311.311	0.00
3801.894	0.00
4365.158	0.00
5011.872	0.00
5754.399	0.00
6606.934	0.00
7585.776	0.00
8709.636	0.00
10000.000	

Operator notes:
