# Supplementary material for: Tailoring the refractive index of impedance-matched ferrite composites
Source: Sci Rep. 2022 Sep 22;12:15818. doi: 10.1038/s41598-022-19188-3 (PMC9500025; doi:10.1038/s41598-022-19188-3)
Supplement: Supplementary file 1 — Supplementary Information 1. [file 41598_2022_19188_MOESM1_ESM.zip › 41598_2022_19188_MOESM1/75-63_Ave.pdf]

MASTERSIZER 2000

MALVERN INSTRUMENTS

Result Analysis Report

Sample Name:
75-63 - Average

SOP Name:
NiZn ferrite

Measured:
21 January 2014 12:39:43

Sample Source & type:
Paris

Measured by:
Mastersizer 2000

Analysed:
21 January 2014 12:39:45

Sample bulk lot ref:
123-ABC

Result Source:
Averaged

Particle Name:
NiZn ferrite

Accessory Name:
Hydro 2000MU (A)

Analysis model:
General purpose

Sensitivity:
Enhanced

Particle RI:
2.730

Absorption:
10

Size range:
0.020 to 2000.000 um

Obscuration:
11.23 %

Dispersant Name:
Water

Dispersant RI:
1.330

Weighted Residual:
0.906 %

Result Emulation:
Off

Concentration:
0.1317 %Vol

Span :
0.795

Uniformity:
0.251

Result units:
Volume

Specific Surface Area:
0.0747 m²/g

Surface Weighted Mean D[3,2]:
80.345 um

Vol. Weighted Mean D[4,3]:
87.742 um

d(0.1): 56.998 um **d(0.5): 83.940 um** **d(0.9): 123.733 um**

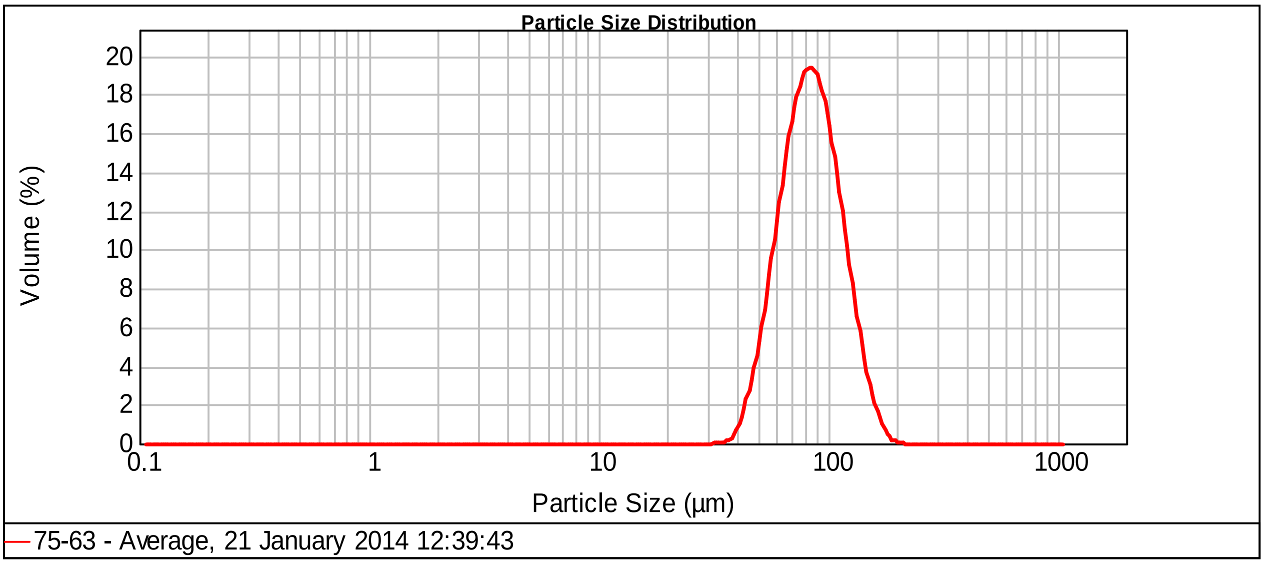

Size (µm)	Volume In %
0.010	0.00
0.011	0.00
0.013	0.00
0.015	0.00
0.017	0.00
0.020	0.00
0.023	0.00
0.026	0.00
0.030	0.00
0.035	0.00
0.040	0.00
0.046	0.00
0.052	0.00
0.060	0.00
0.069	0.00
0.079	0.00
0.091	0.00
0.105	

Size (µm)	Volume In %
0.105	0.00
0.120	0.00
0.138	0.00
0.158	0.00
0.182	0.00
0.209	0.00
0.240	0.00
0.275	0.00
0.316	0.00
0.363	0.00
0.417	0.00
0.479	0.00
0.550	0.00
0.631	0.00
0.724	0.00
0.832	0.00
0.955	0.00
1.096	

Size (µm)	Volume In %
1.096	0.00
1.259	0.00
1.445	0.00
1.660	0.00
1.905	0.00
2.188	0.00
2.512	0.00
2.884	0.00
3.311	0.00
3.802	0.00
4.365	0.00
5.012	0.00
5.754	0.00
6.607	0.00
7.586	0.00
8.710	0.00
10.000	0.00
11.482	

Size (µm)	Volume In %
11.482	0.00
13.183	0.00
15.136	0.00
17.378	0.00
19.953	0.00
22.909	0.00
26.303	0.00
30.200	0.01
34.674	0.17
39.811	1.42
45.709	4.08
52.481	8.17
60.256	12.78
69.183	16.35
79.433	17.52
91.201	15.78
104.713	11.84
120.226	

Size (µm)	Volume In %
120.226	7.27
138.038	3.42
158.489	1.08
181.970	0.12
208.930	0.00
239.883	0.00
275.423	0.00
316.228	0.00
363.078	0.00
416.869	0.00
478.630	0.00
549.541	0.00
630.957	0.00
724.436	0.00
831.764	0.00
954.993	0.00
1096.478	0.00
1258.925	

Size (µm)	Volume In %
1258.925	0.00
1445.440	0.00
1659.587	0.00
1905.461	0.00
2187.762	0.00
2511.886	0.00
2884.032	0.00
3311.311	0.00
3801.894	0.00
4365.158	0.00
5011.872	0.00
5754.399	0.00
6606.934	0.00
7585.776	0.00
8709.636	0.00
10000.000	

Operator notes:
