# Supplementary material for: Tailoring the refractive index of impedance-matched ferrite composites
Source: Sci Rep. 2022 Sep 22;12:15818. doi: 10.1038/s41598-022-19188-3 (PMC9500025; doi:10.1038/s41598-022-19188-3)
Supplement: Supplementary file 1 — Supplementary Information 1. [file 41598_2022_19188_MOESM1_ESM.zip › 41598_2022_19188_MOESM1/93-75_Ave.pdf]

MASTERSIZER 2000

Result Analysis Report

Sample Name:
93-75 - Average

SOP Name:
NiZn ferrite

Measured:
21 January 2014 12:32:16

Sample Source & type:
Paris

Measured by:
Mastersizer 2000

Analysed:
21 January 2014 12:32:17

Sample bulk lot ref:
123-ABC

Result Source:
Averaged

Particle Name:
NiZn ferrite

Accessory Name:
Hydro 2000MU (A)

Analysis model:
General purpose

Sensitivity:
Enhanced

Particle RI:
2.730

Absorption:
10

Size range:
0.020 to 2000.000 um

Obscuration:
9.47 %

Dispersant Name:
Water

Dispersant RI:
1.330

Weighted Residual:
0.987 %

Result Emulation:
Off

Concentration:
0.1273 %Vol

Span :
0.790

Uniformity:
0.249

Result units:
Volume

Specific Surface Area:
0.0647 m²/g

Surface Weighted Mean D[3,2]:
92.721 um

Vol. Weighted Mean D[4,3]:
101.132 um

d(0.1): 65.925 um **d(0.5): 96.749 um** **d(0.9): 142.361 um**

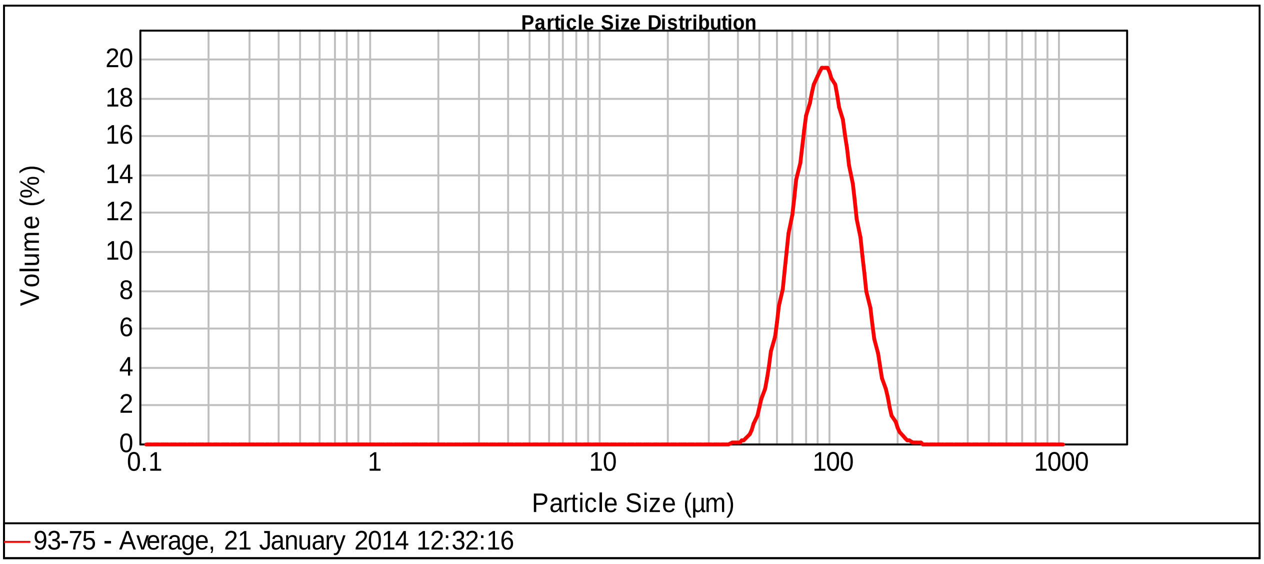

Size (µm)	Volume In %	Size (µm)	Volume In %	Size (µm)	Volume In %	Size (µm)	Volume In %	Size (µm)	Volume In %	Size (µm)	Volume In %
0.010	0.00	0.105	0.00	1.096	0.00	11.482	0.00	120.226	11.99	1258.925	0.00
0.011	0.00	0.120	0.00	1.259	0.00	13.183	0.00	138.038	7.31	1445.440	0.00
0.013	0.00	0.138	0.00	1.445	0.00	15.136	0.00	158.489	3.50	1659.587	0.00
0.015	0.00	0.158	0.00	1.660	0.00	17.378	0.00	181.970	1.07	1905.461	0.00
0.017	0.00	0.182	0.00	1.905	0.00	19.953	0.00	208.930	0.14	2187.762	0.00
0.020	0.00	0.209	0.00	2.188	0.00	22.909	0.00	239.883	0.00	2511.886	0.00
0.023	0.00	0.240	0.00	2.512	0.00	26.303	0.00	275.423	0.00	2884.032	0.00
0.026	0.00	0.275	0.00	2.884	0.00	30.200	0.00	316.228	0.00	3311.311	0.00
0.030	0.00	0.316	0.00	3.311	0.00	34.674	0.00	363.078	0.00	3801.894	0.00
0.035	0.00	0.363	0.00	3.802	0.00	39.811	0.13	416.869	0.00	4365.158	0.00
0.040	0.00	0.417	0.00	4.365	0.00	45.709	1.27	478.630	0.00	5011.872	0.00
0.046	0.00	0.479	0.00	5.012	0.00	52.481	3.91	549.541	0.00	5754.399	0.00
0.052	0.00	0.550	0.00	5.754	0.00	60.256	8.02	630.957	0.00	6606.934	0.00
0.060	0.00	0.631	0.00	6.607	0.00	69.183	12.70	724.436	0.00	7585.776	0.00
0.069	0.00	0.724	0.00	7.586	0.00	79.433	16.41	831.764	0.00	8709.636	0.00
0.079	0.00	0.832	0.00	8.710	0.00	91.201	17.65	954.993	0.00	10000.000	
0.091	0.00	0.955	0.00	10.000	0.00	104.713	15.91	1096.478	0.00		
0.105		1.096		11.482		120.226		1258.925			

Operator notes:
