# Supplementary figures and images for: Tailoring the refractive index of impedance-matched ferrite composites
Source: Sci Rep. 2022 Sep 22;12:15818. doi: 10.1038/s41598-022-19188-3 (PMC9500025; doi:10.1038/s41598-022-19188-3)

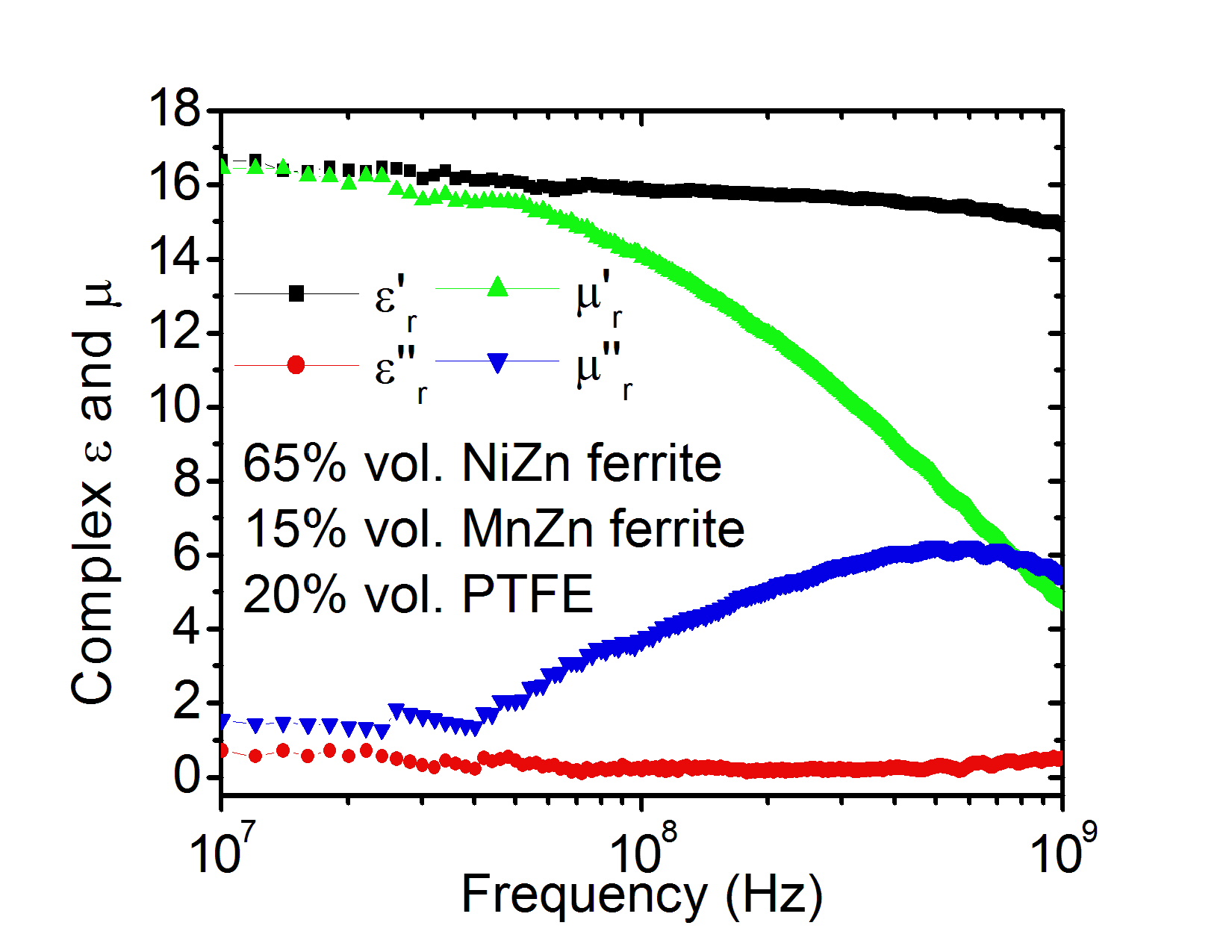

Supplement: Supplementary file 2 — Supplementary Information 2. [file 41598_2022_19188_MOESM2_ESM.jpg]
